# Deutsche Universitäten machen Ergebnisse klinischer Arzneimittelstudien unzureichend öffentlich – Das sollte sich ändern

**DOI:** 10.1007/s00103-020-03246-0

**Published:** 2020-11-11

**Authors:** Peter Grabitz, Till Brückner, Daniel Strech

**Affiliations:** 1 QUEST – Center, Berlin Institute of Health (BIH), Charité – Universitätsmedizin Berlin, Anna-Louisa-Karsch-Str. 2, 10178 Berlin, Deutschland; 2 TranspariMED, Bristol, Großbritannien

**Keywords:** Klinische Studien, Universitätskliniken, Register, Einhaltung von Richtlinien, Retrospektive Erhebung, Publikationsbias, Forschungsethik, Clinical trials, University medical centers, Registries, Guideline adherence, Retrospective study, Publication bias, Research ethics

## Abstract

Ergebnisse klinischer Arzneimittelstudien müssen nach Abschluss zeitnah und nichtselektiv veröffentlicht werden. Die Ergebnispublikation ist als zentrale ethische Regel in der Deklaration von Helsinki des Weltärztebundes festgehalten. Deutsche Universitätskliniken sind vermehrt in die Kritik geraten, diesen Anforderungen nicht ausreichend nachzukommen.

In diesem Beitrag werden verschiedene Publikationsformen von klinischen Arzneimittelstudien (Fachartikel und Kurzberichte) diskutiert und der Status quo der Ergebnispublikation deutscher Universitätskliniken analysiert. Dazu wurden 3 Register und Datenbanken für klinische Studien auf Veröffentlichung von Kurzberichten geprüft: das European Union Clinical Trials Register (EUCTR), das US-amerikanische Register ClinicalTrials.gov und das rein deutschsprachige Portal PharmNet.Bund. Positionen unterschiedlicher Interessengruppen werden zusammengetragen und mögliche Schritte zur Verbesserung vorgeschlagen.

Derzeit erfüllen deutsche Universitätskliniken ihre regulatorischen und ethischen Verpflichtungen im Hinblick auf die Ergebnisveröffentlichung klinischer Arzneimittelstudien nur unzureichend. Zwei Jahre nach Studienende hatten 2 Drittel der Studien, die 2010–2014 abgeschlossen wurden und auf ClinicalTrials.gov verzeichnet sind, noch keine Ergebnisse als Fachartikel veröffentlicht, im Register selbst waren nur für 4,7 % als Kurzberichte hinterlegt. Im EUCTR liegt die Publikationsrate von Kurzberichten bei weniger als 7 %. Die Untersuchung der Datenbank PharmNet.Bund zeigte Ergebnisberichte für weniger als 15 % der dortigen Einträge.

Um das Abschneiden der deutschen Universitätskliniken im Bereich Ergebnispublikation von klinischen Arzneimittelstudien zu verbessern, braucht es politischen Willen und Engagement der Kliniken selbst. Die Veröffentlichung der Ergebnisse aller klinischen Studien ist mit geringen Kosten verbunden. Die Vorteile für öffentliche Gesundheit und Wissenschaft überwiegen bei Weitem den jetzt notwendigen Mehraufwand.

**Zusatzmaterial online:**

Zusätzliche Informationen sind in der Online-Version dieses Artikels (10.1007/s00103-020-03246-0) enthalten.

## Abstract (en)

The results of all clinical drug trials should be published promptly and nonselectively after trial completion. The publication of results appears as a central ethical rule in the Declaration of Helsinki of the World Medical Association. German university hospitals are increasingly being criticized for not meeting these requirements sufficiently.

In this article, different forms of publication of clinical drug trial results are discussed (summary results on registries and journal articles) and the current performance of German university hospitals is analyzed. Three registries and databases for clinical studies were examined for publication of summary results: the European Union Clinical Trials Register (EUCTR), the US registry ClinicalTrials.gov and the exclusively German language portal PharmNet.Bund. Positions of different stakeholders are outlined and possible steps for improvement are proposed.

German university hospitals do not sufficiently fulfil their regulatory and ethical obligations regarding the publication of clinical drug trial results. Two years after the study completion date, two thirds of the studies listed on ClinicalTrials.gov that were completed from 2010–2014 had not yet published results in scientific journals and only 4.7% had posted summary results in the registry. In the European trial registry, the publication rate in the form of summary results has been found to be less than 7%. Less than 15% of relevant entries in the PharmNet.Bund database have results available.

In order to improve the reporting performance of German university hospitals, political will and commitment of the hospitals themselves are needed. The benefits of access to all clinical drug trial results for public health and science far outweigh the (marginal) additional investments that university hospitals have to make to ensure that all their trial results are made public in line with regulatory and ethical requirements.

„Klinische Studien sind ein wesentlicher Schlüssel zum medizinischen Fortschritt.“ So beschreibt der Wissenschaftsrat in einer Stellungnahme die Bedeutung klinischer Studien [1].

Um ihrer Schlüsselrolle beim medizinischen Fortschritt gerecht zu werden, müssen klinische Arzneimittelstudien vor ihrem Start in Register eingetragen werden, denn so bekommen Forschende, ÄrztInnen und PatientInnen Überblick darüber, welche Studien zurzeit laufen oder in der Vergangenheit durchgeführt wurden. Nach Abschluss der Studie müssen die Ergebnisse zeitnah und nichtselektiv veröffentlicht werden. Beides, Registrierung und Ergebnisveröffentlichung, sind deshalb seit 2013 als zentrale ethische Regeln in der Deklaration von Helsinki (Artikel 35 und 36) des Weltärztebundes festgehalten [2].

## Publikation von Fachartikeln in wissenschaftlichen Journalen

Traditionell wird die Ergebnisveröffentlichung als Fachartikel in einem wissenschaftlichen Journal mit Peer-Review-Verfahren realisiert. Eine Studie von Wieschowski et al. (2019) und die dazugehörige interaktive Webseite [3] zeigen für alle 36 deutschen medizinischen Fakultäten, wie oft und wie schnell die Ergebnisse ihrer abgeschlossenen klinischen Studien in Fachzeitschriften auffindbar sind [4]. Als Datengrundlage dienten dabei Studien deutscher Universitätskliniken mit einem Abschlussdatum zwischen 2009 und 2013 im US-amerikanischen Register ClinicalTrials.gov und im Deutschen Register Klinischer Studien (DRKS). In einem mehrstufigen Suchverfahren, das sowohl Identifikationsnummern der Studien als auch Schlagwortkombinationen beinhaltete, wurde versucht, korrespondierende Fachpublikationen zu identifizieren. Zwei Jahre nach Studienende waren für ca. 2 Drittel (63 %) noch keine Ergebnisse in Fachartikeln auffindbar. Für ca. ein Viertel (26 %) der abgeschlossenen klinischen Studien konnten selbst nach 6 und mehr Jahren keine veröffentlichten Ergebnisse gefunden werden. Deutsche Universitätskliniken haben damit Resultate von mindestens 171 klinischen Studien mit mindestens 18.000 PatientInnen, die in den Jahren 2010–2014 abgeschlossen wurden, nicht veröffentlicht.

## Publikation von Kurzberichten in Registern

Die Weltgesundheitsorganisation (WHO) empfiehlt zusätzlich zur Deklaration von Helsinki in einem gemeinsamen Statement mit mehr als 20 Forschungsförderern, dass eine Zusammenfassung der Ergebnisse jeder Studie innerhalb eines Jahres nach Studienabschluss in dem jeweiligen Register publik gemacht werden sollte [5].

Für die klinischen Studien der deutschen Universitätskliniken sind im Besonderen 4 verschiedene Register bzw. Datenbanken relevant: das European Union Clinical Trials Register (EUCTR), ClinicalTrials.gov, das Deutsche Register Klinischer Studien (DRKS) und das rein deutschsprachige Portal PharmNet.Bund.

Interventionelle klinische Prüfungen mit Arzneimitteln in der Europäischen Union müssen in der Datenbank *EudraCT (European Union Drug Regulating Authorities Clinical Trials)* eingetragen werden und sind öffentlich im *EUCTR* einsehbar. Studienergebnisse sollten dort gemäß rechtlichen Vorgaben innerhalb von maximal 12 Monaten nach Studienende in strukturierter Form als Kurzberichte bereitgestellt werden (siehe auch Leitlinie 2012/C 302/03 der EU-Kommission).

Für die meisten anderen klinischen Studien benutzen deutsche Universitätskliniken zudem *ClinicalTrials.gov* und das *DRKS*; beide sind international anerkannt. Das DRKS akzeptiert alle Arten von klinischen Studien, wobei hier keine Kurzberichte publiziert werden, sondern nur Fachartikel als Studienergebnisse verlinkt werden. In ClinicalTrials.gov können Sponsoren ebenfalls alle Arten von Studien registrieren und Ergebnisse lassen sich in strukturierter Form als Kurzberichte hochladen. Außerdem gibt es noch das deutsche Portal *PharmNet.Bund*, das international und auch national außerhalb der Fachkreise für klinische Forschung kaum bekannt ist.

Der rechtliche Hintergrund für die Publikation von Kurzberichten in Registern ist komplex und wird in mehreren EU-weiten Richt- und Leitlinien sowie Verordnungen und im deutschen Arzneimittelgesetz (AMG) und der Good-Clinical-Practice-(GCP-)Verordnung festgelegt (siehe Infobox 1 für weitere Definitionen und Erläuterungen).

Universitätskliniken sind (gemäß §13 Absatz 8–9 GCP-Verordnung, §42b AMG) dazu verpflichtet, für interventionelle klinische Prüfungen mit Arzneimitteln in der Europäischen Union einen Ergebnisbericht innerhalb von maximal 12 Monaten an die jeweils zuständige Bundesoberbehörde zu schicken. Diese prüft die Ergebnisse, speist sie in PharmNet.Bund ein und leitet sie als zusammenfassende Ergebnisse an das Europäische Register EUCTR weiter (gemäß §14 Absatz 3 Nummer 7 GCP-Verordnung, §42b AMG).

Von besonderer Relevanz ist, dass in Deutschland (anders als in einigen anderen EU-Ländern) europäische Anforderungen umgesetzt wurden, indem eine zusätzliche nationale Datenbank – PharmNet.Bund – erstellt wurde. Die Verantwortung zur Publikation im europäischen Register liegt laut GCP-Verordnung in Deutschland bei der zuständigen Bundesoberbehörde (nicht beim Sponsor!). Die Schnittstelle zwischen deutschem und europäischem Register wird von den zuständigen Bundesoberbehörden jedoch nicht bedient. Zudem war in unterschiedlichen Fachkreisen strittig, ob die oben genannte europäische *Leitlinie 2012/C 302/03 *vollumfänglich greift.

Vielen Sponsoren klinischer Studien wurde daher erst im Juni 2019 bewusst, dass sie selbst für das Reporting auf EudraCT zuständig sind, als das Bundesinstitut für Arzneimittel und Medizinprodukte (BfArM) ein Schreiben veröffentlichte mit dem Ziel, „alle Sponsoren von in der EU durchgeführten klinischen Studien an ihre Verpflichtung [zu] erinnern, Zusammenfassungen der Ergebnisse abgeschlossener Studien in der Datenbank der EU für klinische Studien öffentlich zugänglich zu machen.“ Hier stellte das BfArM klar: „Die Einreichung der Ergebnisse bei der EudraCT-Datenbank liegt in der direkten Verantwortung der Sponsoren“ [6].

Wir haben untersucht, wie oft Kurzberichte zu den von deutschen Universitätskliniken in den 3 international anerkannten Registern und der deutschen Datenbank verzeichneten klinischen Studien veröffentlicht wurden bzw. auffindbar sind. Die *EUCTR-Daten* wurden aus dem Report von TranspariMED und der BUKO-Pharma-Kampagne vom Dezember 2019 entnommen. Sie basieren auf handgeprüften Ergebnissen des „EU Trials Trackers“ [7], der sich wiederum direkt aus den Registereintragungen des EUCTR speist. Die Daten *aus ClinicalTrials.gov* wurden der bereits erwähnten Website entnommen [3]. Über die Optionen „Published within 24 months“ und „Registry=CT.gov“ wurde nach Kurzberichten im Register gesucht. Wieschowski et al. haben in einem mehrstufigen Verfahren ebenfalls nach Volltextpublikationen von klinischen Studien deutscher Universitäten auf ClinicalTrials.gov und DRKS gesucht.

Bei *PharmNet.Bund* wurde im Februar 2019 die Identifikation der klinischen Prüfungen pro Universitätsklinikum über das Feld B.1.1. „Name des Sponsors“ mit Städtenamen in unterschiedlicher Schreibform und vor- und nachgestellten Fragezeichen (z. B. ?Muenster?/?Münster?) vorgenommen. Zudem erfolgte eine zusätzliche Suche mit aktiviertem Feld „Are results available?“. Alle weiteren Felder verblieben mit „no restrictions“ markiert. Daraus ergibt sich der Anteil an Studien mit publizierten Ergebnisreports.

## Verpflichtungen nicht erfüllt

**Figure 1:**
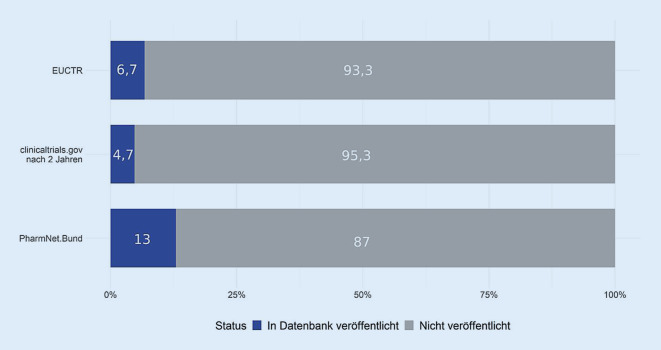

Abb. 1 zeigt als Ergebnis der Auswertung, dass die deutschen Universitätskliniken ihre diversen regulatorischen und ethischen Verpflichtungen im Hinblick auf die Ergebnisveröffentlichung in den oben genannten Registern/Datenbanken derzeit nur sehr unzureichend erfüllen. Die Tatsache, dass deutsche Universitätskliniken im Durchschnitt nur für knapp 7 % der von ihnen durchgeführten klinischen Prüfungen Kurzberichte im europäischen Register EUCTR veröffentlichen (lediglich die Universität Münster sticht mit 61 % positiv heraus [8]), sorgte seit Dezember 2019 für viel mediale Berichterstattung. Ein Report von TranspariMED und der BUKO-Pharma-Kampagne hatte darauf aufmerksam gemacht [9, 10]. Zudem zeigt Abb. 1, dass nach 2 Jahren lediglich 58 von 1224 CT.gov-Studien (4,7 %) Daten als Kurzberichte hochgeladen haben, 266 der 1224 Studien (21,7 %) verlinken eine korrekte Volltextpublikation im gleichen Zeitraum. Durch die zusätzlich angewandten Suchen von Wieschowski et al. ließen sich zwar für 451 von 1224 Studien (36,8 %) nach 2 Jahren Volltextergebnisse identifizieren (nicht dargestellt). Es bleiben damit jedoch noch immer knapp 2 Drittel der Studien nach 2 Jahren ohne auffindbare Ergebnisse.

Das BfArM, das Institut für Qualität und Wirtschaftlichkeit im Gesundheitswesen (IQWiG), der Marburger Bund – Verband der angestellten und beamteten Ärztinnen und Ärzte Deutschlands e. V. und die Cochrane Library haben in der Folge deutsche Universitätskliniken zur Ergebnisveröffentlichung aufgerufen (Stellungnahmen in Infobox 2).

Im Vergleich zu Universitäten schneiden die großen forschenden Arzneimittelhersteller viel besser ab [9, 11, 12]. Auf dem europäischen Register EUCTR haben die entsprechenden Hersteller meist Veröffentlichungsraten von 90–100 %.

### PharmNet.Bund: Fragliche Relevanz

In einem Kommentar des KKS-Netzwerks (Koordinierungszentren für Klinische Studien; [13]), der nach Rücksprache mit den beiden Bundesoberbehörden BfArM und Paul-Ehrlich-Institut (PEI) erfolgte, wurde darauf hingewiesen, dass die Veröffentlichungen von Kurzberichten nach aktueller Rechtslage nicht in EUCTR, sondern in PharmNet.Bund realisiert werden. Abb. 1 zeigt jedoch, dass auch auf PharmNet.Bund der Großteil der klinischen Prüfungen von Universitätskliniken keine Ergebnisberichte vorweist (siehe auch Online-Zusatzmaterial mit einer Aufschlüsselung nach Universitätskliniken. Quelle: eigene Recherche im Februar 2019).

Es ist im Einzelnen nicht genau nachvollziehbar, warum auch auf PharmNet.Bund nur in sehr begrenztem Maß Ergebnisberichte klinischer Prüfungen veröffentlicht werden. Denkbar wäre sowohl, dass Sponsoren ihrer Verpflichtung aus §13 Absatz 9 GCP-Verordnung und §42b AMG nicht nachkommen und nicht in allen Fällen Ergebnisberichte an die Bundesoberbehörden schicken, als auch, dass die Bundesoberbehörden selbst durch einen Rückstau an zu prüfenden Ergebnisberichten zur Unvollständigkeit der PharmNet.Bund-Datenbank beitragen. Die praktische Relevanz der Veröffentlichung von Kurzberichten auf PharmNet.Bund ist aber ohnehin fraglich, da es kein international anerkanntes Register ist. Zudem ist die deutsche Datenbank wenig bekannt, schwer zu durchsuchen und für nichtdeutschsprachige Interessierte nicht zugänglich.

## Ergebnisse in Studienregistern erfüllen eine wichtige Funktion

Die Veröffentlichung von gesetzlich geregelten und qualitätsgesicherten Kurzberichten in Studienregistern dient PatientInnen und ÄrztInnen als wichtige Informationsquelle. Weiterhin können diese Kurzberichte die Erarbeitung und Aktualisierung von systematischen Übersichtsarbeiten, klinischen Leitlinien und Nutzenbewertungen erleichtern.

Eine ergänzende Ergebnisveröffentlichung sind die klassischen Fachartikel in begutachteten Fachzeitschriften. Diese betten Studienergebnisse in bestimmte klinische und wissenschaftliche Kontexte ein. Die zeitliche Verzögerung, mit der Publikationen in Fachzeitschriften erfolgen, stellt aber ein erhebliches Problem für die klinische Praxis, die Forschung und für die PatientInnen dar. Die Veröffentlichungen in Registern bieten zudem die Vorteile, dass sie – anders als viele wissenschaftliche Fachjournale – öffentlich einsehbar sind und keine Bezahlschranke (Paywall) aufweisen.

## Vorstoß der WHO ohne deutsche Beteiligung

Auch die WHO hat bereits im Jahr 2015 klar Stellung zur Ergebnispublikation von Studien bezogen. Es sei „unethisch Forschung am Menschen durchzuführen, ohne die Ergebnisse dieser Forschung zu veröffentlichen und zu verbreiten. Insbesondere das Zurückhalten von Ergebnissen kann zukünftige StudienteilnehmerInnen einem unnötigen Risiko aussetzen“ [14].

Im Jahr 2017 haben über 20 große Forschungsförderer eine WHO-geführte gemeinsame Erklärung zu wünschenswerten Praktiken der Publikation und Registrierung von klinischen Studien unterzeichnet [5] und verpflichteten sich, Richtlinien für korrekte Registrierung und Veröffentlichung von Ergebnissen klinischer Studien zu entwickeln und umzusetzen. Der britische Wellcome Trust setzt in seiner Förderrichtlinie beispielsweise fest, dass alle Ergebnisse und Daten von geförderten klinischen Studien innerhalb von 12 Monaten nach Studienende im jeweiligen Register veröffentlicht werden müssen [15]. Sollten die Sponsoren der geförderten Studien diesen Regeln nicht folgen, können Fördergelder zurückgehalten oder zukünftige Förderungen ausgeschlossen werden [16]. Die beiden großen deutschen Förderer für klinische Studien, die Deutsche Forschungsgemeinschaft (DFG) und das Bundesministerium für Bildung und Forschung (BMBF), gehören bislang nicht zu den Unterzeichnenden der WHO-Erklärung.

## Warum veröffentlichen Universitätskliniken keine Resultate in Registern?

Nicht selten wird als Begründung für die unzureichende Veröffentlichung auf technische Barrieren verwiesen und das europäische Register ist nachgewiesenermaßen nicht nutzerfreundlich. Biomedizinische Forschung sieht sich jedoch häufig mit technischen Herausforderungen konfrontiert. Im Vereinigten Königreich (UK), wo die Rate an Ergebnispublikationen ähnlich schlecht war, konnten signifikante Verbesserungen erzielt werden, nachdem auch politischer Druck ausgeübt wurde. Das QUEST Center am Berlin Institute of Health der Charité hat im September 2019 einen Workshop mit deutschen Studienzentren durchgeführt, um die technischen Probleme anzugehen und Lösungen zu erarbeiten [17]. Seither hat die EMA den Prozess zudem weitergehend vereinfacht.

## Was muss passieren?

### Politischer Wille

Im Vereinigten Königreich wurden klare Erfolge erzielt, indem sich das Wissenschaftskomitee des britischen Parlaments für die Sache stark gemacht hat. Es bleibt zu überlegen, ob auch deutsche Akteure ähnliche Schritte gehen können. Das Ergebnis der Aktivitäten des britischen Parlamentes wird eindrucksvoll in einer Videoaufzeichnung zur Anhörung von medizinischen Fakultäten dargestellt [18].

### Engagement der Universitätskliniken

Über die in diesem Artikel dargestellte mangelnde Veröffentlichungsrate in der Datenbank PharmNet.Bund ließe sich weitergehend diskutieren. Haben Universitätskliniken die Ergebnisberichte der von ihnen durchgeführten klinischen Prüfungen nicht an die Bundesoberbehörden weitergeleitet? Oder sind vor allem die Bundesoberbehörden ihrer Pflicht nicht nachgekommen, die Ergebnisberichte in die Datenbank hochzuladen? Letztlich wird die Klärung des Sachverhaltes aber nur geringfügigen Mehrwert für die Zukunft bringen, denn die europäische Zulassungsbehörde EMA arbeitet bereits seit einigen Jahren daran, das Zugangsportal und die Datenbank sowie Mechanismen für klinische Studienpublikation in der EU zu erneuern und europaweit zu vereinheitlichen. Mit Inkrafttreten des in *§66 EU-Verordnung 536/2014* [19] vorgesehenen Portals um Ende 2021 werden Sponsoren (und damit die Universitätskliniken selbst) für die Veröffentlichung von Ergebnissen rechtlich bindend verantwortlich sein. Die Verordnung sieht in Artikel 94 zudem explizit Sanktionen auf nationaler Ebene vor.

Häufig argumentieren Universitätskliniken, dass sie keine neuen Prozesse anstoßen wollen, bevor sich das System sowieso erneuert. Fest steht jedoch: Aus ethischer Perspektive ist es inakzeptabel, dass Studienergebnisse gar nicht oder erst mit großem zeitlichen Verzug veröffentlicht werden.

Universitäten sollten auch unabhängig von rechtlichen Anforderungen ihrem ethischen Auftrag gerecht werden und Studienergebnisse zeitnah veröffentlichen. Hierfür setzt sich auch das KKS-Netzwerk ein und bündelte Hintergrundinformationen und eigene Expertise in einem Faktenblatt zum Thema „Veröffentlichung von Ergebnissen klinischer Studien“ [20].

Universitäten sollten schon jetzt einen Überblick über die noch zu publizierenden Studien gewinnen (in naher Zukunft müssen sie es sowieso!), wenn nötig Personal einstellen, Prozesse für Registrierung bündeln und Materialien erstellen, um es StudienleiterInnen einfacher zu machen, Resultate hochzuladen. Dass diese Schritte zur Verbesserung führen, konnte im Vereinigten Königreich gezeigt werden. Die Expertise von mehr als 15 erfolgreichen europäischen Universitätskliniken wurde vor Kurzem in einem Manual gebündelt [21]. Das Rezept ist also vorhanden.

Die Veröffentlichung der Ergebnisse aller klinischen Studien in Registern wird mit vergleichsweise wenig Kosten verbunden sein, denn die Verbreitung von Forschungsergebnissen ist nur ein geringer Bestandteil der Gesamtkosten für die Durchführung klinischer Studien. Die Vorteile für öffentliche Gesundheit und Wissenschaft selbst – zusammen mit der Notwendigkeit, ethische Anforderungen zu erfüllen – überwiegen bei Weitem den jetzt notwendigen Mehraufwand. Update im Oktober 2020: In den ersten neun Monaten des Jahres 2020 haben deutsche Universitätskliniken 154 Ergebnisse klinischer Studien auf EUCTR veröffentlicht, ein deutlicher Anstieg gegenüber den nur 32 Ergebnissen, die in den fünf Jahren zuvor hochgeladen wurden. Einige Universitätskliniken scheinen auf dem Weg zu sein, alle ausstehenden Ergebnisse klinischer Prüfungen im EUCTR zu ergänzen [7]. Im Fall von PharmNet.Bund gab es im August 2020 eine Antwort auf eine Anfrage nach Informationsfreiheitsgesetz, in der berichtet wurde, dass ein Großteil der zu prüfenden Ergebnisberichten bearbeitet wurde [22]. Inwieweit die Anzahl von Ergebnisberichten zu abgeschlossenen klinischen Studien auch außerhalb der stark regulierten Arzneimittelstudien zunimmt, wird gegenwärtig in einem Update der bereits zitierten Querschnittsstudie von Wieschowski et al. untersucht [4].

#### Infobox 1 Rechtliche Hintergründe und Begriffe

- Klinische (Arzneimittel‑)Prüfung und klinische (Arzneimittel‑)Studie
  Der Begriff *klinische Studie* bezieht sowohl interventionelle als auch beobachtende Studien mit ein. Eine *klinische (Arzneimittel‑)Prüfung* ist wiederum eine interventionelle klinische Studie, die außerdem unter die Definition in §4 Abs. 23 AMG fällt.
- Für klinische Prüfungen mit Arzneimitteln, die in der europäischen Union durchgeführt werden, findet sich die Verpflichtung, Ergebnisse zu publizieren, in der *Richtlinie 2001/20/EG* [23], auf die zudem weitere später verabschiedete Verordnungen Bezug nehmen (*Verordnung (EG) Nr. 726/2004* [24], *Verordnung (EG) Nr. 1901/2006 *[25]). Diese **Richtlinie** wurde in Deutschland durch das *Arzneimittelgesetz (AMG)* umgesetzt sowie durch die untergeordnete *Good-Clinical-Practice(GCP)-Verordnung* konkretisiert.
- Zudem sieht **Leitlinie** *2012/C 302/03 *[26] vor, dass für klinische Prüfungen mit Arzneimitteln, die in der europäischen Union durchgeführt werden, innerhalb von 12 Monaten nach Studienende Resultate ins EUCTR hochgeladen werden sollten.
- Die unterschiedlichen einzelstaatlichen Umsetzungen der *Richtlinie 2001/20/EG* hat die EU-Kommission schließlich bewogen, im Jahr 2014 die *EU-Verordnung 536/2014* [19] zu erlassen.
- Diese ist 2014 in Kraft getreten, wird jedoch erst 6 Monate nach der Fertigstellung eines neuen EU-weiten Portals anwendbar. Dessen Veröffentlichung war zunächst für 2016 geplant, verzögerte sich aber. Das Inkrafttreten wird derzeit um Ende 2021 erwartet.
- Spätestens wenn dieses vorgesehene EU-Portal an den Start geht, wird die Rolle der Ergebnispublikation vereinheitlicht und klar bei den Sponsoren selbst liegen (laut *EU-Verordnung 536/2014 Art. 37 Abs. 4*). Die Verordnung sieht in Artikel 94 zudem explizit Sanktionen auf nationaler Ebene vor.

#### Infobox 2 Stellungnahmen zur Ergebnisveröffentlichung von klinischen Studien

Das *Bundesinstitut für Arzneimittel und Medizinprodukte (BfArM):* „Eine unzureichende Berichterstattung im Allgemeinen und eine selektive Berichterstattung über klinische Prüfungen mit positivem Ergebnis kann zu potenziell vermeidbaren Redundanzen bei der Durchführung klinischer Prüfungen führen und die ökonomische und wissenschaftliche Effizienz klinischer Forschung beeinträchtigen. Außerdem können unveröffentlichte klinische Prüfungen mit ungünstigem Ergebnis negative Auswirkungen auf die öffentliche Gesundheit haben“ [6].

*Institut für Qualität und Wirtschaftlichkeit im Gesundheitswesen (IQWIG)*: Prof. Dr. med. Jürgen Windeler, Leiter IQWIG, spricht sich klar für Konsequenzen aus: Öffentliche Förderung solle davon abhängig gemacht werden, ob vorher durchgeführte Projekte in vorgeschriebener Weise veröffentlicht worden sind [27].

*Marburger Bund:* Bundesvorsitzende Dr. Susanne Johna: „Die Ergebnisse klinischer Forschung müssen öffentlich zugänglich sein. Es ist völlig inakzeptabel, wenn Universitäten dieser Verpflichtung zur Transparenz nur unzureichend nachkommen“ [28].

Die Chefredakteurin der *Cochrane* Library, Karla Soares-Weiser: „Die Verfügbarkeit von Daten aus klinischen Studien ist für Cochrane unerlässlich, um qualitativ hochwertige und relevante systematische Reviews erstellen zu können. Andernfalls riskieren wir, dass sich unsere Reviews nur auf einen Teil der Daten stützen. Das könnte dazu führen, dass der Nutzen einer bestimmten Gesundheitsmaßnahme überbewertet wird oder dass mögliche Schäden unterschätzt werden“ [29].

## Caption Electronic Supplementary Material


