# Supplementary material for: The publication of clinical trial results by German universities is insufficient—this should change
Source: Bundesgesundheitsblatt Gesundheitsforschung Gesundheitsschutz. 2020 Nov 11;63(12):1531–7. [Article in German] doi: 10.1007/s00103-020-03246-0 (PMC7686199; doi:10.1007/s00103-020-03246-0)

Bundesgesundheitsblatt 12/2020

Elektronisches Zusatzmaterial zum Beitrag:

Deutsche Universitäten machen Ergebnisse klinischer Arzneimittelstudien unzureichend öffentlich – Das sollte sich ändern.

Peter Grabitz[1], Till Brückner[1,2], Daniel Strech[1]

[1] QUEST Center for Transforming Biomedical Research, Berlin Institute of Health (BIH), Charité - Universitätsmedizin Berlin, Berlin, Deutschland
[2] TranspariMED, Bristol, UK

Korrespondenzautor:
Peter Grabitz,
QUEST–Center, Berlin Institute of Health (BIH),
Charité-Universitätsmedizin Berlin,
Anna-Louisa-Karsch-Str. 2,
10178 Berlin, Deutschland
peter.grabitz@charite.de

Inhalt:

Abb. Z1: Relativer Anteil veröffentlichter Ergebnisreports für klinische Prüfungen in PharmNet.Bund pro Universitätsklinikum. Quelle: Eigene Recherche im Februar 2019

Abb. Z2: Anzahl veröffentlichter und nicht veröffentlichter Ergebnisreports von klinischen Prüfungen in PharmNet.Bund pro Universitätsklinikum. Quelle: Eigene Recherche im Februar 2019

Abb. Z1: Relativer Anteil veröffentlichter Ergebnisreports für klinische Prüfungen in PharmNet.Bund pro Universitätsklinikum. Quelle: Eigene Recherche im Februar 2019

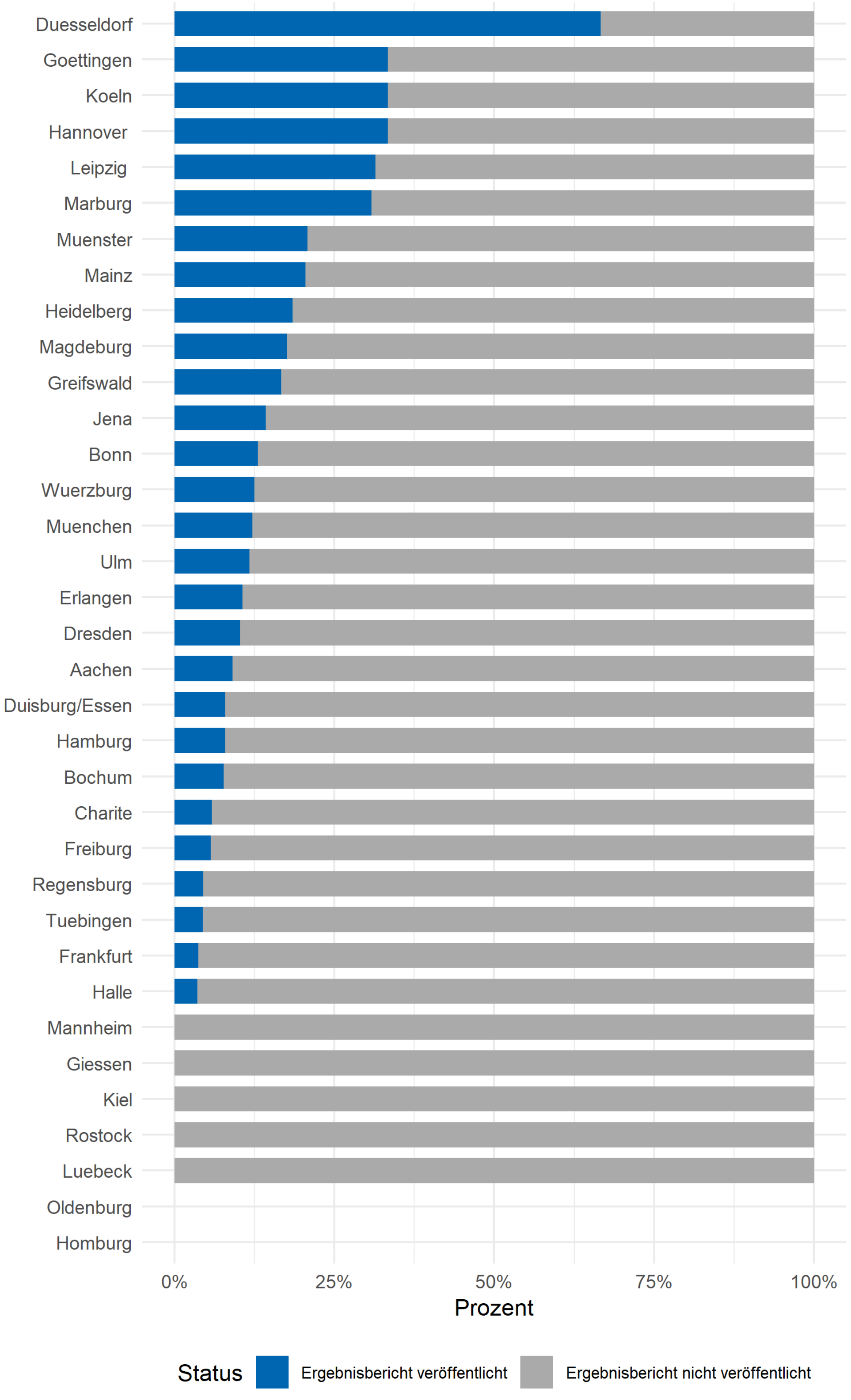

Abb. Z2: Anzahl veröffentlichter und nicht veröffentlichter Ergebnisreports von klinischen Prüfungen in PharmNet.Bund pro Universitätsklinikum. Quelle: Eigene Recherche im Februar 2019

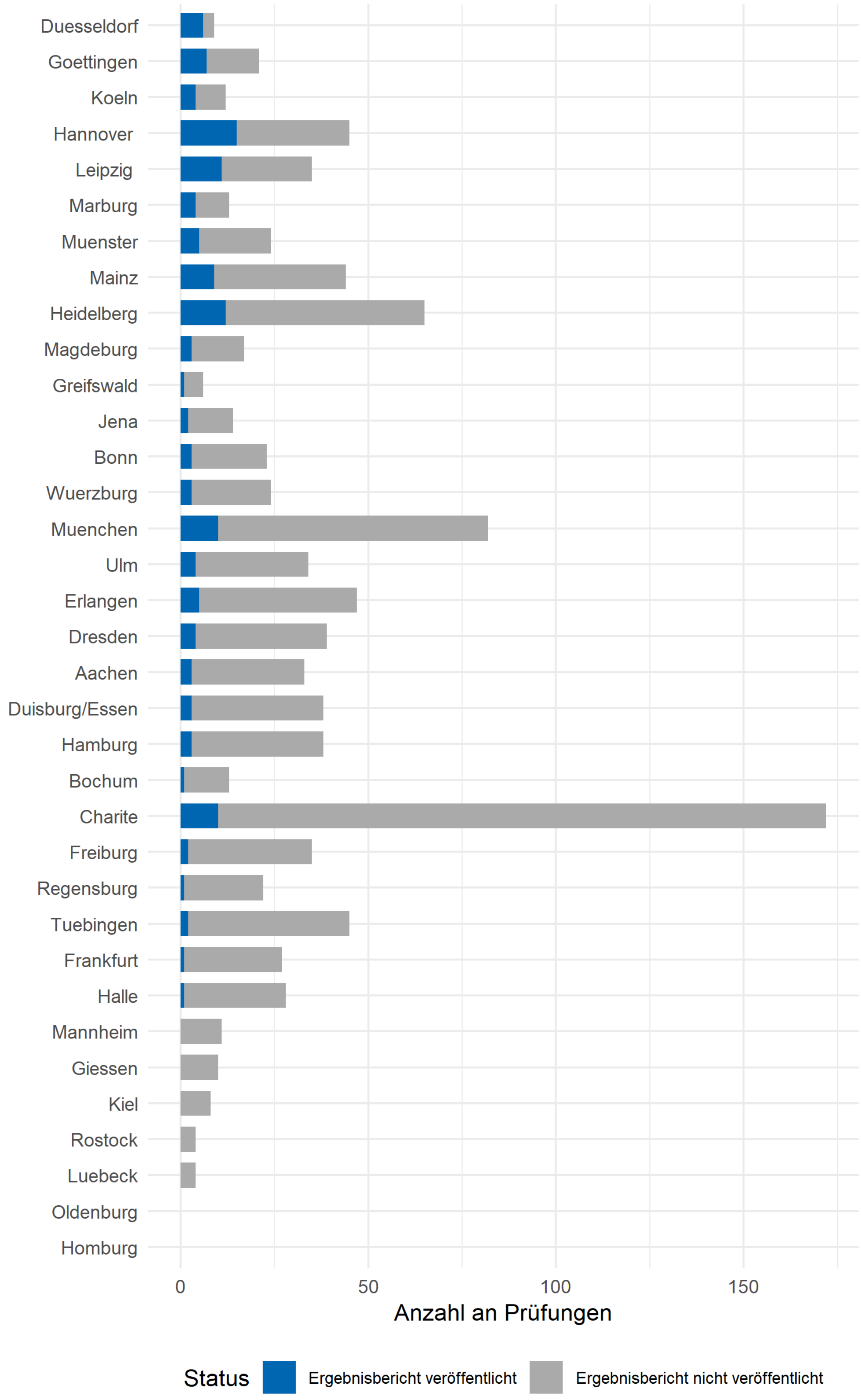

Supplement: Supplementary file 1 [file 103_2020_3246_MOESM1_ESM.pdf]
